# A Stackelberg Game-Based Caching Incentive Scheme for Roadside Units in VANETs

**DOI:** 10.3390/s20226625

**Published:** 2020-11-19

**Authors:** Yang Wang, Yuankun Lin, Lingyu Chen, Jianghong Shi

**Affiliations:** School of Informatics, Xiamen University, Xiamen 361005, China; yangwang@stu.xmu.edu.cn (Y.W.); 23320171153126@stu.xmu.edu.cn (Y.L.); shijh@xmu.edu.cn (J.S.)

**Keywords:** vehicular ad hoc networks, Stackelberg game, caching scheme, pricing strategy

## Abstract

As a key technology of intelligent transportation systems (ITS), vehicular ad hoc networks (VANETs) have been promising to provide safety and infotainment for drivers and passengers. To support different applications about traffic safety, traffic efficiency, autonomous driving and entertainment, it is important to investigate how to effectively deliver content in VANETs. Since it takes resources such as bandwidth and power for base stations (BSs) or roadside units (RSUs) to deliver content, the optimal pricing strategy for BSs and the optimal caching incentive scheme for RSUs need to be studied. In this paper, a framework of content delivery is proposed first, where each moving vehicle can obtain small-volume content files from either the nearest BS or the nearest RSU according to the competition among them. Then, the profit models for both BSs and RSUs are established based on stochastic geometry and point processes theory. Next, a caching incentive scheme for RSUs based on Stackelberg game is proposed, where both competition sides (i.e., BSs and RSUs) can maximize their own profits. Besides, a backward introduction method is introduced to solve the Stackelberg equilibrium. Finally, the simulation results demonstrate that BSs can obtain their own optimal pricing strategy for maximizing the profit as well as RSUs can obtain the optimal caching scheme with the maximum profit during the content delivery.

## 1. Introduction

In recent years, as a key technology of intelligent transportation systems (ITS), vehicular ad hoc networks (VANETs) have caught much attention because of the enormous potential in improving road safety and traffic efficiency as well as in providing drivers and passengers with infotainment services [1,2]. In order to support these applications, numerous roadside units (RSUs) need to be deployed along the road to boost network capacity rather than just the existing cellular network coverage. Therefore, two main communication types, vehicle-to-vehicle (V2V) and vehicle-to-infrastructure (V2I), are adopted to support different applications in VANETs [3,4,5,6]. Usually, moving vehicles can download the requested content from RSUs or other wireless access nodes with cached replicas of requested content through V2I communication rather than fetch the content from the remote server, which can significantly reduce the download latency and the data traffic in VANETs [7,8]. Moreover, V2V communication can be used to achieve reliable safety applications [9].

However, the content in VANETs usually changes over time, and it is often location-related and delay-limited, such as vehicle state information [10], warning messages [11], and autonomous driving services [12,13,14]. Therefore, the vehicles must obtain the content needed in an extremely short time, otherwise it is likely to become invalid and outdated information for moving vehicles. Therefore, how to effectively deliver content for providing the safety and pleasant drive becomes a critical issue in VANETs [8]. In fact, caching content in the edge nodes (e.g., RSUs) of the network can reduce the congestion of the backhaul network and the download latency, and then motivate the content downloads from moving vehicles [14]. However, the edge nodes in VANETs have limited cache space and need to pay the running cost [8,15]. Therefore, the optimal caching strategy developed for them is a key issue for content caching.

In the last few years, a lot of research work about content caching in VANETs has been carried out [8,15,16,17,18,19,20,21,22,23,24,25,26]. Wang, X. takes advantage of vehicular cloud to reduce the content delivery cost and latency, where vehicular cloud members store and provide the content locally so that vehicles can rapidly retrieve the content from the nearest member [16]. However, the authors assume that the channel is perfect and ignore the timeliness of content. In [17], Sun, Y. proposes a cooperative downloading scheme based on vehicular mobility prediction for reducing downloading latency, where RSUs can deliver content to vehicles with low cost. However, the delay of fetching content from base stations (BSs) is ignored and the channel is assumed ideal. In order to minimize downloading delay, Ndikumana, A. et al. propose a deep learning-based caching, where caching decisions depend on passengers’ features by deploying the multi-access edge computing servers at RSUs [18]. However, high computational complexity of the proposed scheme will increase the running cost of RSUs and the channel fading is also ignored. In [19], based on mobility prediction and social attributes, Yao, L. proposes a cooperative caching scheme where a caching node sharing more social attributes with the content requester is more likely to be interested in the same contents and delivery the contents to others with similar interests. However, the channel is still assumed to be ideal. Moreover, on the assumption of ideal channel, Liu, T. et al. exploit the moving information of vehicles to maximize the successful content download probability in [20]. Therefore, with considering the pass loss and multi-path fading, Rahim, M. et al. present a content caching policy based on Gale Shapley stable matching algorithm to make the vehicles maximize the data download rate and reduce the information retrieval delay [21]. However, the timeliness of the content is ignored. Since game theory has been widely used for cost optimization of content caching and delivery [22], or for joint optimization of cost and transmission capacity [23,24], some game models are adopted to analyze the optimal pricing and caching schemes. In [23], an edge computing-based vehicular content dissemination framework is developed and the system is modeled as a first-price sealed auction game, where the edge computing device can select the optimal vehicle which satisfy its requirement to relay the content for more efficiently content delivery and more profit. However, the timeliness of content is not considered. In [24], the heterogeneous vehicular network is modeled as a coalition formation game, where vehicles are grouped into coalitions based on their interests and requests and vehicles in the same coalition can download their requested content by downloading from RSUs, BSs, or other vehicles to achieve the minimum download cost. However, the competition among vehicles in the coverage of the RSU is not mentioned and the timeliness of content is also ignored. Wang, S. et al. propose a vehicular edge computing caching scheme that leverages idle storage resources of parked vehicles and then present a content placement algorithm based on an iterative ascending price auction game [25]. However, the auction process can cause additional latency in a dense vehicular network. Li, J. et al. propose a bargaining game-based pricing model to motivate the vehicles and RSUs to act more positively in the content delivery for lower service cost and more profits, respectively [26]. However, the ideal channel is assumed and the timeliness of content is ignored, while in [8], a pricing model for content delivery is proposed based on a Stackelberg game, where the RSU and parked vehicles compete for moving vehicles to obtain the profits. However, it has high complexity and the timeliness of content is ignored. Analogously, a pricing scheme based on the Stackelberg game is also proposed to model the interaction between two moving vehicles in [15], where a moving vehicle can obtain a part of the content from the BS and the remaining content from a neighboring vehicle with lowest price. However, a moving vehicle as the service provider without the required content must download the content from the BS first, which will increase the download latency of the vehicle with service request.

To the best of our knowledge, few of the existing works consider the impacts of the pricing strategies of different network operators and the caching schemes on the operator selection strategy of moving vehicles. In fact, the competitiveness of different network operators is different and how to formulate the optimal pricing strategy to attract moving vehicles for maximizing their own profits is an important issue of concern for them. Pricing too low will make their own profits thin, or even make them lose money. On the contrary, too high pricing will deter moving vehicles, and thus results in losing profits. In this paper, a game model based on [27] is proposed to describe the interactions between different players (i.e., BSs and RSUs) to obtain the optimal pricing strategy of BSs and the optimal caching scheme of RSUs. Besides, the scheme involving the vehicles selecting which operator to serve is investigated. Moreover, suitable mathematical models of BSs and RSUs are introduced to describe the distributions of BSs and RSUs. Based on this, the coverage probabilities of the networks formed by each of them (homogeneous network) or by both of them (heterogeneous network) are analyzed.

The main contributions of the paper can be threefold.

1. We propose a Stackelberg game-based caching incentive scheme for the RSU in VANETs. Both BS and RSU can deliver content to moving vehicles alone to obtain profit. The moving vehicles can make a choice of downloading content from BS or RSU based on the received signal-to-interference-plus-noise ratio (SINR).
2. Based on the Stackelberg game, a model is given to show the interaction among the BS and the RSU. A backward introduction method is introduced to solve the Stackelberg equilibrium. Based on the established profit models of both BS and RSU, we first solve the optimal caching scheme of RSU w.r.t. the price determined by the BS. Then, based on the scheme, we solve the optimal pricing strategy of the BS. Finally, the Stackelberg equilibrium solution can be obtained.
3. The operation state adjustment scheme of RSUs within a day is designed. Based on the above, work and the running cost of RSUs is further considered. After analyzing the established one-hour profit model of RSUs, the optimal activity density of RSUs at different hours can be obtained.

The rest of this paper is organized as follows. Section 2 describes the system model. The proposed caching incentive scheme of RSUs is analyzed in detail in Section 3. The operation state adjustment for RSUs is given in Section 4. Section 5 gives the simulation results and discussions. Finally, Section 6 concludes the paper.

## 2. System Model

**Figure 1:**
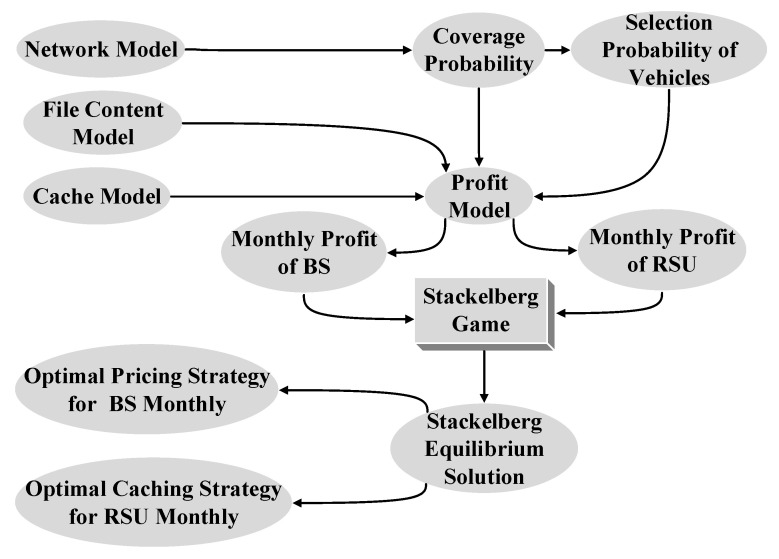
Structure diagram of the proposed caching incentive scheme.

We consider a heterogeneous network consisting of BSs and RSUs. Since the assumptions that the distributions of RSUs (e.g., uniform distribution [17], grid distribution [20]) in some existing models make the analysis not accurate enough, we assume that BSs and RSUs are arranged according to two-dimensional (2D) homogeneous Poisson point process (PPP) and one-dimensional (1D) homogeneous PPP, respectively, based on stochastic geometry theory [28] and point process theory [29]. Then, the coverage probability of effective service of the heterogeneous network is derived. Subsequently, we design a caching incentive scheme based on the Stackelberg game for RSUs. In order to obtain the Stackelberg equilibrium solution, we first establish the profit functions of BS and RSU, respectively. Second, we analyze the concavity and convexity of the profit functions of BSs and RSUs and solve the extreme values to obtain the optimal pricing strategy for the BS and the optimal caching scheme for the RSU. Finally, the Stackelberg equilibrium solution of the game model can be obtained. The structure diagram of the proposed caching incentive scheme is as shown in Figure 1. 

### 2.1. Network Model

**Figure 2:**
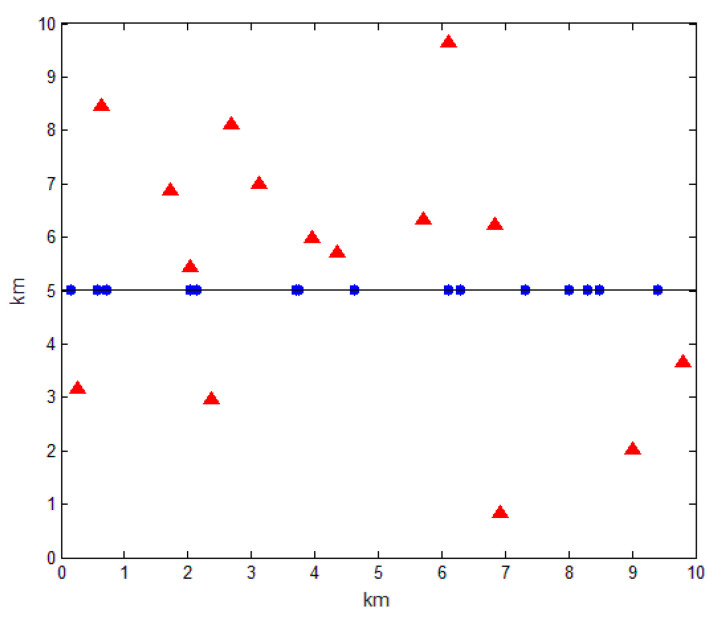
Position distribution diagram of base units (BSs) and roadside units (RSUs).

In the Euclidean plane, there is a heterogeneous network with BSs submitting to 2D homogeneous PPP of intensity $\lambda_{b}$. Moreover, in the plane, there is one straight way, where RSUs obey 1D homogeneous PPP of intensity $\lambda_{r}$. It is assumed that the location distributions of BSs and RSUs are independent of each other, and the communication channels used by the BS and the RSU are different. Therefore, the BS and the RSU will not interfere with each other, and thus the cellular network and the VANET constitute a heterogeneous vehicular network. Here, a simple diagram is drawn as shown in Figure 2, where the red triangle and blue circle represent BS and RSU, respectively, and the line connecting the blue circles represents a straight road. 

Assume that each moving vehicle is only associated with its nearest BS or RSU and it is in coverage if the received SINR at the tagged moving vehicle from the BS or the RSU is larger than some threshold T. Assume that the transmission power of each RSU is $P_{0}$, that of BS is $P_{1}$, and the channel is Rayleigh fading. Then, the received SINR from RSUs at the tagged moving vehicle can be expressed as [30]

(1) $$SINR_{r}=\frac{h_{r}r^{-\alpha }}{I_{r}+\sigma^{2}}$$

where $h_{r}$ follows an exponential distribution with mean $P_{0}$, r is the distance from the tagged vehicle to the nearest RSU, α is the path loss exponent, and $\sigma^{2}=B_{0}\cdot N_{0}$ is the noise power ($B_{0}$ is the bandwidth and $N_{0}$ is the power spectral density of noise). $I_{r}$ is the cumulative interference power received from the other RSUs, which can be expressed as $I_{r}=\underset{i\in \phi /r_{o}}{\sum }g_{i}R_{i}^{-\alpha }$, where ϕ and $\phi /r_{o}$ denote the set of all RSUs with and without the nearest one of tagged vehicle, respectively, $g_{i}$ is the fading coefficient, and $R_{i}$ represents the distance between the *i*-th interfering RSU and the tagged vehicle.

Similarly, the received SINR from BSs at the tagged moving vehicle can be expressed as $SINR_{b}=\frac{h_{b}r^{-\alpha }}{I_{b}+\sigma^{2}}$, where $h_{b}$ follows an exponential distribution with mean $P_{1}$. $I_{b}$ is the cumulative interference power received from the other BSs.

### 2.2. File Content Model

Suppose that there is a large set of content files stored in the BSs’ back-end file server denoted by $F=\left\{F_{1},F_{2},\ldots ,F_{F}\right\}$, where the total number of small-volume files is F and the size of each file is L. The vehicle sends a request independently to download file $F_{i}$ (i=1,2,…,F) with probability $p_{i}$ ($\sum_{i=1}^{F}p_{i}=1$), and the higher ranking of a content file, the greater the requested probability. Since Zipf distribution can approximatively reflect the distribution of different file popularity in the network, the popularity of the *i*-ranked content file, i.e., $p_{i}$, can be modeled as [31]

(2) $$p_{i}=\frac{1/i^{\Omega }}{\sum_{j=1}^{F}1/j^{\Omega }},\;\;\;\;1\leq i\leq F$$

where Ω≥0 reflects the skew of the content popularity. The larger Ω means that the popularity is more uneven, and when Ω=0, the popularity of the files is uniform. In this section, let Ω=1, i.e., the popularity of files obeys standard Zipf distribution.

### 2.3. Caching Model

**Figure 3:**
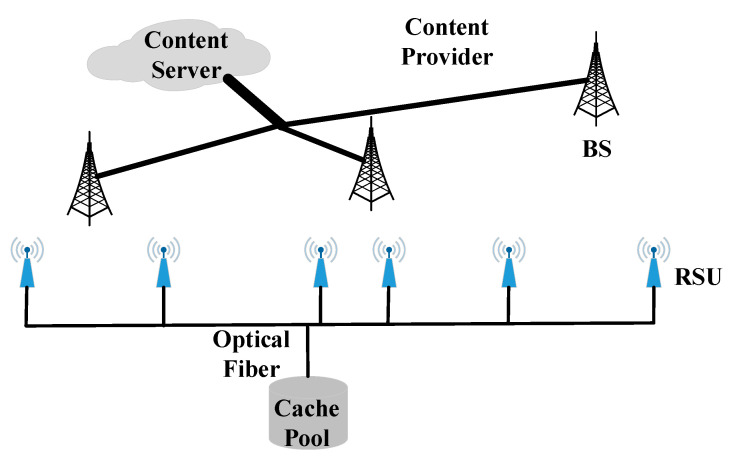
Schematic diagram of caching systems for BSs and RSUs.

The schematic diagram of caching systems for BSs and RSUs is shown in Figure 3. The operator of cellular network has all F files and BSs are connected via a backhaul network, and each BS is connected to the content server. Besides, moving vehicles within the coverage of the cellular network can communicate with the nearest BS to download any files of interest. In a region, the operator of RSUs has a cache pool with sufficient cache space, and the RSUs are connected with each other and all RSUs can link to the cache pool by fiber optic. Since all RSUs are connected to the same pool by optical fiber, they are all able to access the content purchased by any RSU. That is, the RSU can access the content from the cache pool or purchase the content needed from the BS if necessary and capable. Assume that the transmission delay between the cache pool and the RSU is small enough, the moving vehicle with download requests can communicate with the nearest RSU to obtain the files of interest from the cache pool. Besides, the operator of RSUs buys the files from the operator of cellular network as required. 

### 2.4. Effective Service Coverage Probability

As assumed before that each moving vehicle only communicates with the nearest BS or RSU, who can offer reliable services if the received SINR from BSs or RSUs at the concerned vehicle is large enough. Here, the coverage probability of effective service (hereinafter referred to as coverage probability) is used to denote the probability that a moving vehicle can be provided with reliable service by BS or RSU, which can be defined as

(3) $$P_{b}\left(T,\lambda_{b},\alpha \right)≜P_{b}[SINR>T]$$

where T is the SINR threshold of service coverage (SINR threshold for short), $\lambda_{b}$ is the density of BSs, and α is the path loss exponent. Likewise, the coverage probability of RSU can be defined by the same way, i.e., $P_{r}\left(T,\lambda_{r},\alpha \right)≜P_{r}[SINR>T]$, where $\lambda_{r}$ is the density of RSUs. 

#### 2.4.1. Coverage Probabilities of Cellular Network and VANET 

If the location distribution of BSs submits to a 2D homogeneous PPP and the channel between the moving vehicle and the BS is Rayleigh fading, the coverage probability of the cellular network can be calculated by [30]

(4) $$P_{b}\left(T,\lambda_{b},\alpha \right)=\pi \lambda_{b}\int_{0}^{\infty }e^{-\pi \cdot \lambda_{b}\left[1+\rho \left(T,\alpha \right)\right]v-\mu_{b}T\sigma^{2}v^{\alpha /2}}dv$$

where $\rho \left(T,\alpha \right)=T^{2/\alpha }\int_{T^{-2/\alpha }}^{\infty }\frac{1}{1+u^{\alpha /2}}du$, $\mu_{b}=\frac{1}{P_{1}}$ is the reciprocal of BS’s transmission power, and $\sigma^{2}$ is the noise power. When the BS density $\lambda_{b}$ is large enough, the noise can be ignored and then the coverage probability can be simplified to [30]

(5) $$P_{b}\left(T,\lambda_{b},\alpha \right)=\frac{1}{1+\rho \left(T,\alpha \right)}=\frac{1}{1+T^{{}^{\alpha /2}}\int_{T^{{}^{-\alpha /2}}}^{\infty }\frac{1}{1+u^{\alpha /2}}du}$$

In order to calculate the coverage probability of the heterogeneous network consisting of BSs and RSUs, the coverage probability of the VANET consisting of RSUs need to be derived. Assume that the distance between the tagged vehicle and its nearest RSU is r, which follows an exponential distribution with mean λ (because the vehicle location obeys 1D PPP), i.e., the probability density function of r is

(6) $$f_{r}\left(r\right)=\lambda e^{-\lambda r}$$

Then, the coverage probability of RSU can be expressed as

(7) $$P_{r}\left(T,\lambda_{r},\alpha \right)=E_{r}\left[P\left[SINR>T|r\right]\right]=\int_{0}^{\infty }P\left[SINR>T|r\right]f_{r}\left(r\right)dr$$

Substitute Equations (1) and (6) into (7), Equation (7) can be rewritten as

(8) $$\begin{array}{ll} P_{r}\left(T,\lambda_{r},\alpha \right) & =\int_{0}^{\infty }P\left[\frac{h_{r}r^{-\alpha }}{\sigma^{2}+I_{r}}>T|r\right]\lambda_{r}e^{-\lambda_{r}\cdot r}dr\\ & =\int_{0}^{\infty }e^{-\lambda_{r}\cdot r}\cdot P\left[h_{r}>Tr^{\alpha }\left(\sigma^{2}+I_{r}\right)|r\right]\lambda_{r}dr \end{array}$$

Let the inverse of transmission power of RSU be $\mu_{r}$, then the random variable $h_{r}$ follows an exponential distribution with mean $\mu_{r}$, denoted as $h\sim exp\left(\mu_{r}\right)$. Therefore,

(9) $$\begin{array}{ll} P\left[h_{r}>Tr^{\alpha }\left(\sigma^{2}+I_{r}\right)|r\right] & =E_{I_{r}}\left[P\left[h_{r}>Tr^{\alpha }\left(\sigma^{2}+I_{r}\right)|r,I_{r}\right]\right]\\ & =E_{I_{r}}\left[exp\left(-\mu_{r}Tr^{\alpha }\left(\sigma^{2}+I_{r}\right)\right)|r)\right]\\ & =e^{-\mu_{r}Tr^{\alpha }\sigma^{2}}L_{I_{r}}\left(\mu_{r}Tr^{\alpha }\right) \end{array}$$

where $L_{I_{r}}\left(s\right)$ is the Laplace transformation of random variable $I_{r}$. Substitute (9) into (8), Equation (8) can be rewritten as

(10) $$P_{r}\left(T,\lambda_{r},\alpha \right)=\int_{0}^{\infty }e^{-\lambda_{r}\cdot r}\cdot e^{-\mu_{r}Tr^{\alpha }\sigma^{2}}L_{I_{r}}\left(\mu_{r}Tr^{\alpha }\right)\lambda_{r}dr$$

Let $s=\mu_{r}Tr^{\alpha }$ in Equation (A3) of Appendix A, the following equation can be obtained.

(11) $$\begin{array}{ll} L_{I_{r}}\left(\mu_{r}Tr^{\alpha }\right) & =exp\left(-\lambda_{r}\int_{r}^{\infty }\left(1-\frac{\mu_{r}}{\mu_{r}+\mu_{r}Tr^{\alpha }v^{-\alpha }}\right)dv\right)\\ & =exp(-\lambda_{r}\int_{r}^{\infty }\frac{1}{1+{\left(\frac{v}{r}T^{-\frac{1}{\alpha }}\right)}^{\alpha }}dv) \end{array}$$

Let $x=\frac{v}{r}T^{-\frac{1}{\alpha }}$, then

(12) $$L_{I_{r}}\left(\mu_{r}Tr^{\alpha }\right)=exp\left(-\lambda_{r}\cdot rT^{\frac{1}{\alpha }}\int_{T^{-\frac{1}{\alpha }}}^{\infty }\left(\frac{1}{1+x^{\alpha }}\right)dx\right)$$

Substitute Equation (12) into (10), Equation (10) can be expressed as

(13) $$P_{r}\left(T,\lambda_{r},\alpha \right)=\int_{0}^{\infty }exp\left(-\lambda_{r}\cdot r\left[1+T^{\frac{1}{\alpha }}\int_{T^{-\frac{1}{\alpha }}}^{\infty }\left(\frac{1}{1+x^{\alpha }}\right)dx\right]-\mu_{r}Tr^{\alpha }\sigma^{2}\right)\lambda_{r}dr$$

If the interference power is much greater than the noise power (when the RSU density $\lambda_{r}$ is large enough), the influence from the noise can be ignored. Therefore, Equation (13) can be rewritten as

(14) $$\begin{array}{ll} P_{r}\left(T,\lambda_{r},\alpha \right) & =\int_{0}^{\infty }exp\left(-\lambda_{r}\cdot r\left[1+T^{\frac{1}{\alpha }}\int_{T^{-\frac{1}{\alpha }}}^{\infty }\left(\frac{1}{1+x^{\alpha }}\right)dx\right]\right)d\left(\lambda_{r}\cdot r\right)\\ & =\frac{1}{1+T^{\frac{1}{\alpha }}\int_{T^{-\frac{1}{\alpha }}}^{\infty }\left(\frac{1}{1+x^{\alpha }}\right)dx} \end{array}$$

From the above Equations (5) and (14), when the density of access nodes (e.g., BSs and RSUs) submitting to PPP distribution is large enough, the coverage probability of the network is independent of the density value.

#### 2.4.2. Coverage Probability of the Heterogeneous Network 

Suppose that any moving vehicle on the road covered by the BS is termed event A and covered by the RSU is termed event B. Since the location distributions of BSs and RSUs are independent, events A and B are also independent. According to the definition of coverage probability, the occurring probability of event A is $P_{b}\left(T,\lambda_{b},\alpha \right)$ and that of event B is $P_{r}\left(T,\lambda_{r},\alpha \right)$. Since events A and B are independent, the probability that event A does not occur but event B occurs is $\left[1-P_{b}\left(T,\lambda_{b},\alpha \right)\right]P_{r}\left(T,\lambda_{r},\alpha \right)$. Therefore, the probability that the moving vehicle is in the coverage of the heterogeneous network (i.e., the coverage probability of the heterogeneous network) can be expressed as

(15) $$P_{br}\left(T,\lambda_{b},\lambda_{r},\alpha \right)=P_{b}\left(T,\lambda_{b},\alpha \right)+\left[1-P_{b}\left(T,\lambda_{b},\alpha \right)\right]P_{r}\left(T,\lambda_{r},\alpha \right)$$

As seen from the above equation, the coverage probability of the heterogeneous network is larger than that of cellular network or VANET, i.e., $P_{br}\left(T,\lambda_{b},\lambda_{r},\alpha \right)>P_{b}\left(T,\lambda_{b},\alpha \right)$ and $P_{br}\left(T,\lambda_{b},\lambda_{r},\alpha \right)>P_{r}\left(T,\lambda_{r},\alpha \right)$. Then, substitute Equations (5) and (14) into (15) and ignore the noise, the coverage probability can be rewritten as

(16) $$\begin{array}{l} P_{br}\left(T,\alpha \right)=\left[1-\frac{1}{1+T^{2/\alpha }\int_{T^{-2/\alpha }}^{\infty }\frac{1}{1+u^{\alpha /2}}du}\right]\cdot \frac{1}{1+T^{1/\alpha }\int_{T^{-1/\alpha }}^{\infty }\left(\frac{1}{1+x^{\alpha }}\right)dx}\\ \;+\frac{1}{1+T^{2/\alpha }\int_{T^{-2/\alpha }}^{\infty }\frac{1}{1+u^{\alpha /2}}du} \end{array}$$

#### 2.4.3. Operator Selection of Moving Vehicles

Assume that the BS has all content files and the tagged vehicle wants to download file $F_{i}$. If the RSU has cached $F_{i}$, both the BS and the RSU can provide the vehicle with this file. As a result, the vehicle should make a choice between the BS and the RSU. In fact, the selection probability of the vehicle can be calculated based on the coverage probability. For simplicity, assume that the BS charges the vehicle the same as the RSU. Therefore, the received SINR is the only factor influencing the vehicle’s choice, because the received SINR values from BSs and RSUs are different for the vehicles in different locations. Since the vehicle needs to download the small-volume content file, the instantaneous received SINR from BSs or RSUs is concerned rather than the average SINR. Hence, if the instantaneous received SINR from BSs is larger than that from RSUs, the vehicle chooses the nearest BS; otherwise, it chooses the nearest RSU.

Suppose the received SINR from the BS is t (a random variable). According to the definition of coverage probability $P_{b}\left(t,\lambda_{b},\alpha \right)$, the cumulative distribution function of t can be expressed by

(17) $$F_{b}\left(t\right)=P\left[SINR_{BS}<t\right]=\;1-P\left[SINR_{BS}>t\right]=\;1-P_{b}\left(t,\lambda_{b},\alpha \right)$$

Therefore, the probability that the received SINR from BS is t, i.e., the probability density function of t, can be expressed by

(18) $$f_{b}\left(t\right)=F_{b}^{\prime }\left(t\right)={\left[1-P_{b}\left(t,\lambda_{b},\alpha \right)\right]}^{\prime }$$

Similarly, the probability that the received SINR from the RSU is less than t is given by

(19) $$F_{r}\left(t\right)=P\left[SINR_{RSU}<t\right]=1-P_{r}\left(t,\lambda_{r},\alpha \right)$$

Therefore, the probability that the instantaneous received SINR from BSs is larger than that from RSUs at the vehicle can be expressed as

(20) $$p_{BR}=P\left[SINR_{BS}>SINR_{RSU}\right]=\int_{0}^{\infty }f_{b}\left(t\right)\cdot F_{r}\left(t\right)dt=\int_{0}^{\infty }{\left[1-P_{b}\left(t,\lambda_{b},\alpha \right)\right]}^{\prime }\cdot \left[1-P_{r}\left(t,\lambda_{r},\alpha \right)\right]dt$$

Here, let the noise power be $\sigma^{2}=0$ (when density is large enough, noise power can be omitted) and the path loss exponent be α=4. Then, substituting Equations (5) and (14) into (20), $\;p_{BR}$ can be expressed as

(21) $$\begin{array}{ll} p_{BR} & =\int_{0}^{\infty }{\left[1-P_{b}\left(t,\lambda_{b},4\right)\right]}^{\prime }\cdot \left[1-P_{r}\left(t,\lambda_{r},4\right)\right]dt\\ & =\int_{0}^{\infty }(1-\frac{1}{1+\sqrt{t}\int_{\frac{1}{\sqrt{t}}}^{\infty }\frac{1}{1+u^{2}}du}){}^{\prime }\cdot (1-\frac{1}{1+t^{\frac{1}{4}}\int_{t^{-\frac{1}{4}}}^{\infty }\frac{1}{1+u^{4}}du})dt\approx 0.29 \end{array}$$

Assume that the vehicle must be able to obtain the interested content in the network. Since the vehicle chooses either the nearest BS or the nearest RSU, the probability that the instantaneous received SINR from RSUs is larger than that from BSs at the vehicle is

(22) $$p_{RB}=P\left[SINR_{RSU}>SINR_{BS}\right]=1-p_{BR}\approx 0.71$$

Note that when calculating the operator selection probability of the vehicle based on the coverage probability, the densities of BSs and RSUs are supposed to be large enough. As a result, the selection probability of the vehicle is a specific value independent of the noise and the densities of BSs or RSUs.

## 3. Caching Incentive Scheme of RSU

### 3.1. Stackelberg Game

**Figure 4:**
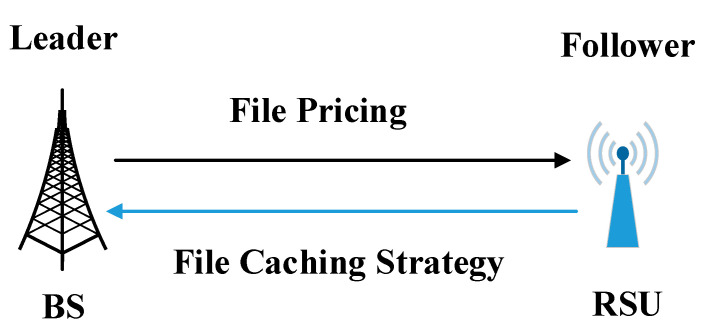
Game model between BS and RSU.

For simplicity, assume that the BSs have all the content files. In order to reduce the network load, improve quality of service, and attract more vehicles to download files, a scheme is designed to encourage the RSUs to cache files. The proposed scheme can effectively motivate the RSU operator to buy and cache files from the BS. In the scheme, the BS prices individual file and charges the RSU operator by the amount of files, and the RSU operator needs to decide how many files to buy and to be stored in the cache pool. Then, the RSU offers download service to the vehicles and charges the vehicles for benefit. As a result, thanks to the reduced network load, BSs can lower the costs of power and bandwidth. As the designer of the caching incentive scheme, the BS has the right to take priority action to design a file pricing strategy. Then, the RSU develops a file caching strategy by the price to decide the number of files to buy. Based on the heterogeneous relationship between BS and RSU, the Stackelberg game is adopted to describe the interaction between these two parties. The Stackelberg game is a commonly used strategic game that consists of leaders and followers competing with each other for certain resources [22]. In this game model, the BS is the leader and the RSU is the follower, and the former moves first and the latter moves subsequently. In order to maximize its own profit, the BS first determines the price of single file. According to the above price, the RSU evaluates whether caching the file can bring profit, and then develops the optimal content caching scheme for maximizing the profit. The game model is shown in Figure 4. 

### 3.2. Profit Models of BS and RSU 

In this section, like before, assume that path loss exponent is α=4 and the densities of BSs and RSUs are large enough so that the noise can be ignored. Therefore, the coverage probability is only related to the threshold T and the operator selection probability of the vehicle is constant. Since the profit of the BS consists of two parts, i.e., the profit from selling content files to the RSU and that from providing the moving vehicles with file download service, which can be calculated by

(23) $$U_{b}\left(C_{f}\right)=C_{f}hL+p_{BR}C_{v}KP_{br}\left(T\right)L\overset{h}{\underset{i=1}{\sum }}p_{i}+C_{v}KP_{b}\left(T\right)L\overset{F}{\underset{i=h+1}{\sum }}p_{i}$$

where $C_{f}\in \left[0,\infty \right)$ is the unit traffic pricing that BS charges the RSU, h is the number of files cached by the RSU representing its caching policy, and L is the size of each file. $p_{i}$ is the popularity of the *i*-ranked content file given by Equation (2) and the probability $p_{BR}$ that the vehicle selects BS is about 0.29 calculated by Equation (21). $C_{v}\in \left[0,\infty \right)$ is the price of single file that the BS charges the vehicle, and the number of download requests is denoted by K. $P_{br}\left(T\right)$ is the coverage probability of the heterogeneous network calculated by Equation (16). In the above Equation (23), the first part is the profit that the BS sells content files to the RSU, the second part is the profit that the BS benefits from the top h files with popularity, and the third part is the profit that the RSU benefits from the rest of files with popularity. The probability that file i requested by the vehicle is $p_{i}$ and F is the amount of all files. Note that $P_{b}\left(T\right)$ is the coverage probability of the cellular network in Equation (5). Therefore, the optimal strategy of the BS, which maximizes the profit function of the BS, can be expressed as

(24) $$C_{f}^{*}=\arg\underset{C_{f}}{max}U_{b}\left(C_{f}\right)$$

The profit function $U_{r}\left(h\right)$ of RSU consists of two parts, i.e., the profit of providing a file download service to the vehicle and the cost of purchasing content files from BS, can be expressed by

(25) $$\begin{array}{c} U_{r}\left(h\right)=\overset{h}{\underset{i=1}{\sum }}\left(C_{v}KP_{br}\left(T\right)p_{RB}Lp_{i}-C_{f}L\right)\\ \;=p_{RB}C_{v}KP_{br}\left(T\right)L\overset{h}{\underset{i=1}{\sum }}p_{i}-C_{f}hL \end{array}$$

where h∈[0,F] is the number of files cached by the RSU, which represents its caching strategy. $p_{RB}$ denotes the probability that the vehicle chooses the RSU, and its value is about 0.71 calculated by Equation (22). In the above Equation (25), the first part is the profit from that the RSU caches these h files and the second part is the cost that the RSU purchases these files from the BS. Therefore, the optimal strategy of the RSU, which maximizes the profit function of the RSU, is given by

(26) $$h^{*}=\arg\underset{h}{max}U_{r}\left(h\right)$$

### 3.3. Stackelberg Equilibrium Solution

Here, a backward induction method is introduced to solve the Stackelberg equilibrium of the above formulated game. The profit function of the RSU is first analyzed, and the caching strategy $h^{*}$ is figured out, which is expressed as the function related to the pricing $C_{f}$ determined by the BS. Second, the profit function of the BS is analyzed and the optimal pricing strategy of the BS $C_{f}^{*}$ is solved based on the precondition that the BS knows the caching strategy of the RSU $h^{*}$. Finally, the Stackelberg equilibrium solution $\left(C_{f}^{*},\;h^{*}\right)$ can be obtained.

#### 3.3.1. Profit Analysis of RSU 

According to Equation (25), the profit of the RSU from the file $F_{i}$ downloaded by the vehicle with probability $p_{i}$ can be expressed as

(27) $$U_{ri}=\left(0.71C_{v}KP_{br}\left(T\right)p_{i}-C_{f}\right)L$$

As seen in (27), whether the RSU caches file $F_{i}$ depends on the positive and negative of $U_{ri}$. If $U_{ri}>0$ (i.e., the file $F_{i}$ can bring profit to the RSU), the RSU will buy $F_{i}$ from the BS and store it in the cache pool. Otherwise, the RSU will not buy file $F_{i}$. Therefore, whether file $F_{i}$ can bring profit to the RSU depends on the probability $p_{i}$, and the RSU only caches the files with higher popularity. 

Since the file popularity submits to standard Zipf distribution (i.e., $p_{i}=\frac{1/i}{\sum_{j=1}^{F}1/j}$), the following expressions can be obtained.

(28) $$\frac{1/\left(h^{*}+1\right)}{\sum_{j=1}^{F}1/j}=p_{h^{*}+1}\leq \frac{C_{f}}{0.71C_{v}KP_{br}\left(T\right)}<p_{h^{*}}=\frac{1/h^{*}}{\sum_{j=1}^{F}1/j}$$

where $h^{*}$ is the optimal strategy of the RSU.

#### 3.3.2. Profit Analysis of BS

In order to figure out the Stackelberg equilibrium solution, the second step of backward induction method is carried out. That is, assume the BS has known that the optimal caching strategy of RSU is $h^{*}$. Substitute $h^{*}$ into the profit function of the BS, the optimal strategy of the BS in equilibrium can be obtained. 

After substituting $h=h^{*}$ into Equation (23), the profit function of the BS can be rewritten as

(29) $$\begin{array}{c} U_{b}\left(C_{f}\right)=C_{f}h^{*}\cdot L+0.29C_{v}KP_{br}\left(T\right)L\overset{h^{*}}{\underset{i=1}{\sum }}p_{i}+C_{v}KP_{b}\left(T\right)L\left(1-\overset{h^{*}}{\underset{i=1}{\sum }}p_{i}\right)\\ \;=C_{f}h^{*}\cdot L+\left[0.29P_{br}\left(T\right)-P_{b}\left(T\right)\right]C_{v}KL\overset{h^{*}}{\underset{i=1}{\sum }}p_{i}+C_{v}KP_{b}\left(T\right)L \end{array}$$

According to Equation (28), when $h^{*}$ remains unchanged, $C_{f}<0.71C_{v}KP_{br}\left(T\right)\frac{1/h^{*}}{\sum_{j=1}^{F}1/j}$. Since only the first term of Equation (29) is related with the pricing strategy of the BS (i.e., $C_{f}$), $C_{f}$ should be as close as possible to $0.71C_{v}KP_{br}\left(T\right)\frac{1/h^{*}}{\sum_{j=1}^{F}1/j}$ for maximizing $U_{b}\left(C_{f}\right)$, which means their difference value $C_{m}$ determined by BS should be small enough. Given the pricing difference (e.g., $C_{m}=10^{-4}$ CNY/MB), the optimal pricing strategy can be obtained as

(30) $$C_{f}^{*}=0.71C_{v}KP_{br}\left(T\right)\frac{1/h^{*}}{\sum_{j=1}^{F}1/j}-C_{m}$$

In consideration of the numerator and denominator of $\overset{h^{*}}{\underset{i=1}{\sum }}p_{i}=\frac{\sum_{i=1}^{h^{*}}1/i}{\sum_{j=1}^{F}1/j}$ are both harmonic numbers, Euler’s constant γ=0.5772156… is introduced, which is defined as [32]

(31) $$\gamma =\underset{n\to \infty }{\lim}\left(H\left(n\right)-\ln n\right)$$

where the *n*-th harmonic number H(n) is expressed as

(32) $$H\left(n\right)=\overset{n}{\underset{j=1}{\sum }}\frac{1}{j}=1+\frac{1}{2}+\frac{1}{3}+\ldots +\frac{1}{n}$$

The greater n means the difference between H(n) and lnn is closer to γ. Since the amount of files F is a large number, the following approximate expression can be obtained.

(33) $$\overset{h^{*}}{\underset{i=1}{\sum }}p_{i}=\frac{\sum_{i=1}^{h^{*}}1/i}{\sum_{j=1}^{F}1/j}\approx \frac{\ln\left(h^{*}\right)+\gamma }{\ln\left(F\right)+\gamma }$$

Therefore, substitute Equations (30) and (33) into (29), Equation (29) can be rewritten as

(34) $$U_{b}=\left(0.71C_{v}KP_{br}\left(T\right)\frac{1}{\sum_{j=1}^{F}1/j}-C_{m}h^{*}\right)L+\left[0.29P_{br}\left(T\right)-P_{b}\left(T\right)\right]C_{v}KL\frac{\ln\left(h^{*}\right)+\gamma }{\ln\left(F\right)+\gamma }+C_{v}KP_{b}\left(T\right)L$$

Then, the first and second order partial derivatives w.r.t. $h^{*}$ of $U_{b}$ can be expressed as

(35) $$\frac{\partial U_{b}}{\partial \left(h^{*}\right)}=-C_{m}L+\frac{\left[0.29P_{br}\left(T\right)-P_{b}\left(T\right)\right]C_{v}KL}{\ln\left(F\right)+\gamma }\cdot {\left(h^{*}\right)}^{-1}$$

(36) $$\frac{\partial^{2}U_{b}}{\partial {\left(h^{*}\right)}^{2}}=-\frac{\left[0.29P_{br}\left(T\right)-P_{b}\left(T\right)\right]C_{v}KL}{\ln\left(F\right)+\gamma }\cdot {\left(h^{*}\right)}^{-2}$$

Next, $U_{b}$ needs to be analyzed based on the positive and negative of $0.29P_{br}\left(T\right)-P_{b}\left(T\right)$.

(1) When $0.29P_{br}\left(T\right)-P_{b}\left(T\right)\leq 0$, $\frac{\partial U_{b}}{\partial \left(h^{*}\right)}<0$ can be obtained. Since $U_{b}$ decreases monotonically w.r.t.$\;h^{*}$, $U_{b}$ can be maximized by $h^{*}=0$. This demonstrates that if the coverage probability of the heterogeneous network is not large enough, it leads to lower the total profit of BS after RSU caches files. Therefore, the BS sets a large value of $C_{f}^{*}$ and forbids the RSU from caching any file.
(2) When $0.29P_{br}\left(T\right)-P_{b}\left(T\right)>0$, $\frac{\partial^{2}U_{b}}{\partial {\left(h^{*}\right)}^{2}}<0$ can be obtained as well as $\underset{h^{*}\to 0}{\lim}\frac{\partial U_{b}}{\partial \left(h^{*}\right)}>0$ and $\underset{h^{*}\to \infty }{\lim}\frac{\partial U_{b}}{\partial \left(h^{*}\right)}=-C_{m}L<0$. Therefore, $U_{b}$ is a strictly concave function w.r.t. $h^{*}$ and the maximum exists. Let $\frac{\partial U_{b}}{\partial \left(h^{*}\right)}=0$, the optimal strategy of RSU is
  (37) $$h^{*}=\frac{\left[0.29P_{br}\left(T\right)-P_{b}\left(T\right)\right]C_{v}K}{\left(\ln\left(F\right)+\gamma \right)C_{m}}$$

Since $h^{*}$ is the number of files that RSU caches, it must be a positive integer. Hence, Equation (37) can be rewritten as

(38) $$h^{*}=\{\begin{array}{cc} \left\lfloor M\right\rfloor , & U_{b}\left(h^{*}=\left\lfloor M\right\rfloor \right)\geq U_{b}\left(h^{*}=\left\lfloor M\right\rfloor +1\right)\\ \left\lfloor M\right\rfloor +1, & U_{b}\left(h^{*}=\left\lfloor M\right\rfloor \right)<U_{b}\left(h^{*}=\left\lfloor M\right\rfloor +1\right) \end{array}$$

where ⌊·⌋ is a flooring integer function and $M=\frac{\left[0.29P_{br}\left(T\right)-P_{b}\left(T\right)\right]}{\left(\ln\left(F\right)+\gamma \right)C_{m}}$. Substituting (38) into (30), the optimal pricing strategy of the BS can be obtained as

(39) $$C_{f}^{*}=\{\begin{array}{cc} 0.71C_{v}KP_{br}\left(T\right)\frac{1/\left\lfloor M\right\rfloor }{\sum_{j=1}^{F}1/j}-C_{m}, & U_{b}\left(h^{*}=\left\lfloor M\right\rfloor \right)\geq U_{b}\left(h^{*}=\left\lfloor M\right\rfloor +1\right)\\ 0.71C_{v}KP_{br}\left(T\right)\frac{1/\left(\left\lfloor M\right\rfloor +1\right)}{\sum_{j=1}^{F}1/j}-C_{m}, & U_{b}\left(h^{*}=\left\lfloor M\right\rfloor \right)<U_{b}\left(h^{*}=\left\lfloor M\right\rfloor +1\right) \end{array}$$

Therefore, when $0.29P_{br}\left(T\right)-P_{b}\left(T\right)>0$, the RSU will cache files. Equations (39) and (38) are the optimal strategies of the BS and the RSU, respectively, i.e., $\left(C_{f}^{*},h^{*}\right)$ is the Stackelberg equilibrium solution. That whether or not $0.29P_{br}\left(T\right)-P_{b}\left(T\right)>0$ depends on the communication requirement (i.e., the threshold T) from moving vehicles.

#### 3.3.3. Changes of File Popularity 

When $0.29P_{br}\left(T\right)-P_{b}\left(T\right)>0$, according to Equation (38), the RSU should cache the top $h^{*}$ files with popularity every month so as to maximize the profit. However, the popularity ranking of files will change as time goes on [33,34]. Therefore, the RSU should adjust the caching content based on the popularity ranking of files every month, and buy and cache the top $h^{*}$ files with popularity from the BS. Then, the RSU can always obtain profit by providing the vehicles with file downloading service.

## 4. Operation State Adjustment for RSUs 

Based on the assumption that the noise can be ignored if the RSU density is large enough, when the coverage probability of the heterogeneous network is large enough to increase the profit of the BS, there exists the optimal $h^{*}$ as shown in Equation (38), i.e., the RSU can cache $h^{*}$ files to maximize the monthly profit. In fact, the RSU needs to pay the running cost and the vehicle density changes in a day. When the number of download requests from the vehicles is low in some period of one day, keeping high density of running RSUs will lower the profit of RSU in this period of time. Therefore, based on these factors, some RSUs should be turned off. Then, the running RSU density decreases as well as the interfering power, but the location of RSUs still obeys 1D PPP. As a result, the noise needs to be taken into account. This section aims at researching on adjusting the operation state of RSUs in a day, i.e., searching the density of active RSUs. 

The one-hour profit function of the RSU in a day is mainly composed of two parts, i.e., the profit of providing the vehicles with file download service and the running cost of the RSU, which can be expressed as

(40) $$U_{r}\left(\lambda_{r}\right)=C_{v}kL\overset{h^{*}}{\underset{i=1}{\sum }}p_{i}\cdot P_{br}\left(T,\lambda_{r}\right)\cdot p_{RB}-C_{r}D\lambda_{r}$$

where $\lambda_{r}$ is the RSU density and $C_{v}$ is the unit file pricing that the RSU charges vehicles. k denotes the number of download requests in an hour by vehicles on the road, L is the size of single file, and $h^{*}$ is the optimal number of files cached by the RSU monthly calculated by Equation (38). Besides, the running cost of single RSU each hour is $C_{r}$ and D is the road length. In Equation (40), the first item denotes the profit that the RSU can obtain in an hour from these $h^{*}$ files cached by itself and the second item is the running cost of the RSU.

In Equation (40), $P_{br}\left(T,\lambda_{r}\right)$ and $p_{RB}$ denote the coverage probability of the heterogeneous network and the probability that the vehicle chooses the RSU, respectively. As long as the BS density is large enough, the coverage probability of the BS has no concern with the density value. While the RSU density decreases, both the coverage probability of the RSU and the probability that the vehicle chooses the RSU will reduce. Therefore, $P_{br}\left(T,\lambda_{r}\right)$ and $p_{RB}$ are related with the RSU density $\lambda_{r}$ and the threshold T. According to Equations (13), (15) and (20), $P_{br}\left(T,\lambda_{r}\right)$ and $p_{RB}$ can be expressed as

(41) $$\begin{array}{l} \;P_{br}\left(T,\lambda_{r}\right)=P_{b}\left(T\right)+\left[1-P_{b}\left(T\right)\right]P_{r}\left(T,\lambda_{r}\right)\\ =P_{b}\left(T\right)+\left[1-P_{b}\left(T\right)\right]\cdot \int_{0}^{\infty }exp\left(-\lambda_{r}\cdot r\left[1+T^{\frac{1}{4}}\int_{T^{-\frac{1}{4}}}^{\infty }\left(\frac{1}{1+x^{4}}\right)dx\right]-\mu_{r}Tr^{4}\sigma^{2}\right)\lambda_{r}dr \end{array}$$

(42) $$p_{RB}=1-\int_{0}^{\infty }{\left[1-P_{b}\left(t\right)\right]}^{\prime }\cdot \left[1-P_{r}\left(t,\lambda_{r}\right)\right]dt$$

Therefore,

(43) $$\begin{array}{l} P_{br}\left(T,\lambda_{r}\right)\cdot p_{RB}\\ =\left[P_{b}\left(T\right)+\left[1-P_{b}\left(T\right)\right]P_{r}\left(T,\lambda_{r}\right)\right]\cdot \left[1-\int_{0}^{\infty }{\left[1-P_{b}\left(t\right)\right]}^{\prime }\cdot \left[1-P_{r}\left(t,\lambda_{r}\right)\right]dt\right]\\ =\left[P_{b}\left(T\right)+\left[1-P_{b}\left(T\right)\right]P_{r}\left(T,\lambda_{r}\right)\right]\cdot \int_{0}^{\infty }f\left(t\right)\cdot P_{r}\left(t,\lambda_{r}\right)dt\\ =\left[P_{b}\left(T\right)+\left[1-P_{b}\left(T\right)\right]P_{r}\left(T,\lambda_{r}\right)\right]\cdot G\left(\lambda_{r}\right) \end{array}$$

where $G\left(\lambda_{r}\right)=\int_{0}^{\infty }f\left(t\right)\cdot P_{r}\left(t,\lambda_{r}\right)dt$ and $f\left(t\right)={\left[1-P_{b}\left(t\right)\right]}^{\prime }$. Then, substitute (43) into (40), $U_{r}\left(\lambda_{r}\right)$ can be written as

(44) $$U_{r}\left(\lambda_{r}\right)=C_{v}kL\overset{h^{*}}{\underset{i=1}{\sum }}p_{i}\cdot \left[P_{b}\left(T\right)+\left[1-P_{b}\left(T\right)\right]P_{r}\left(T,\lambda_{r}\right)\right]\cdot G\left(\lambda_{r}\right)-C_{r}D\lambda_{r}$$

Here, the proof of concavity and convexity of the one-hour profit function of the RSU is given in detail in Appendix B. If the maximum of $U_{r}\left(\lambda_{r}\right)$ exists, the value which makes its first-order derivative zero is the optimal RSU density $\lambda_{r}^{*}$.

## 5. Validation of the Proposed Scheme 

### 5.1. Verification of Expressions of Coverage Probability of RSU and Selection Probability of Vehicle 

In this subsection, in order to show the differences between the theoretical values and the simulation results of coverage probability of RSU, and the impacts from the noise and selection probability of the vehicle with different RSU densities, the Monte Carlo simulation method is used in MATLAB and the simulations are carried out 10^6^ times for the average results. Here, the distribution of BSs is 2D PPP, and the distribution of RSUs is 1D PPP. The simulation parameters are given in Table 1.

**Figure 5:**
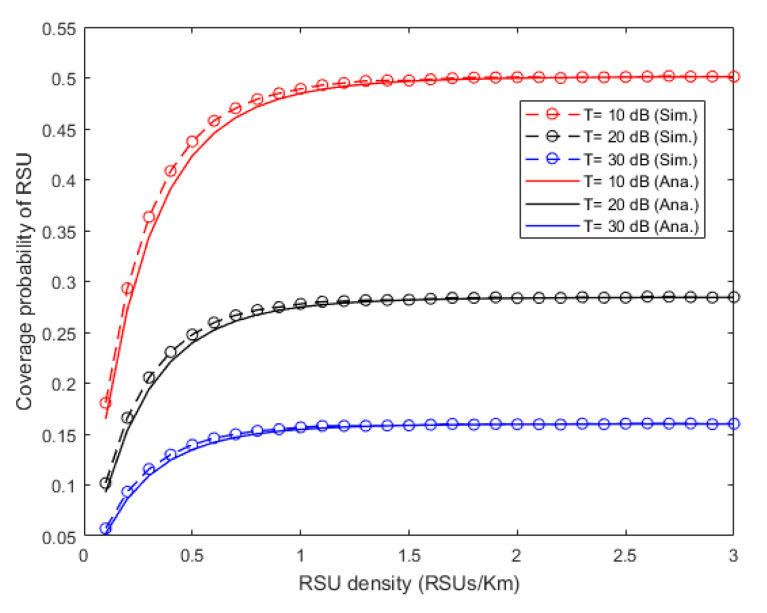
Influence of noise on coverage probability under different coverage thresholds.

The relationships between the coverage probability of RSU and its density are shown in Figure 5, where the theoretical values are calculated by Equation (13). From the figure, the simulation results are very close to the theoretical ones, especially when the RSU density is larger than 1 RSUs/km. Besides, when the RSU density is small, the coverage probability increases with the increase of the RSU density. When the RSU density is large enough, the sum of the interference RSUs is so large that the noise can be ignored. In addition, the coverage probability hardly increases after reaching a certain value. In particular, when the SINR thresholds are 10dB, 20dB, and 30 dB, the upper limits of the curves of coverage probability are 0.501, 0.284, and 0.160, respectively, which are consistent with the calculation results of Equation (14).

**Figure 6:**
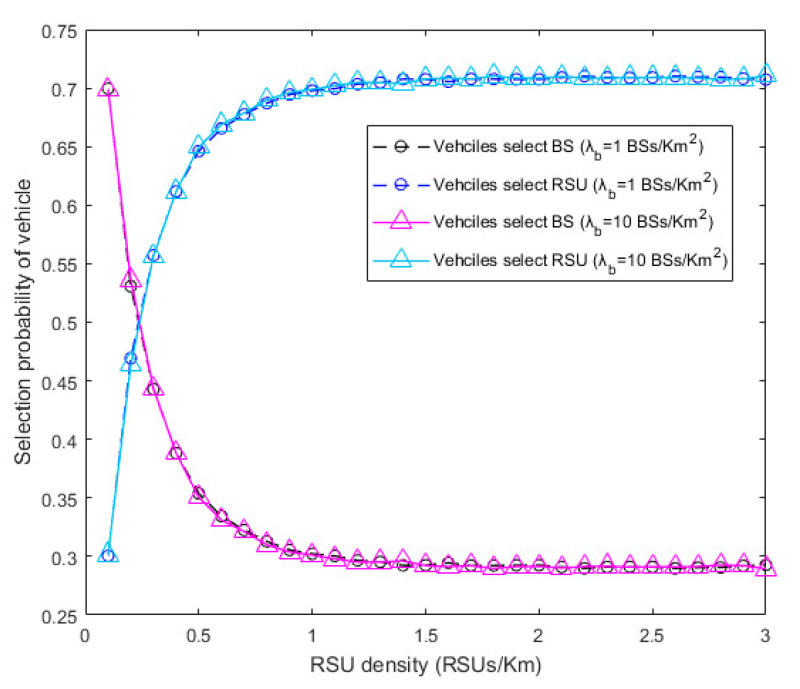
Relationship between selection probability of vehicles and RSU density.

The probabilities that the moving vehicle chooses the BS or the RSU under different RSU densities are given in Figure 6. Since the vehicle can only choose either BS or RSU, the sum of the probabilities of those two choices is 1. The vehicle makes a choice based on the received SINR values from BSs and RSUs. For the vehicle, the probability of choosing the RSU is equivalent to the probability that the instantaneous SINR from RSUs is larger than that from BSs. When the RSU density is small and the BS density is large (the coverage probability of BS is up to upper limit), the channel condition between the vehicle and the RSU is bad and it is easily influenced by the environment. Therefore, the received SINR from the RSU is small, which leads to low probability that the vehicle chooses the RSU. However, as the RSU density increases, so does the probability that the vehicle chooses the RSU. This probability will almost not grow when the coverage probability of RSU reaches the limitation. When the densities of both BSs and RSUs are large enough, the simulation results by which the vehicle chooses the BS and RSU are mostly 0.29 and 0.71, which are coincident with the computation values of Equations (21) and (22), respectively. Though BSs and RSUs both obey PPP distribution, the difference between their spatial distribution dimensions leads to a large probability that the vehicle chooses RSU. As seen from the figure, in the case that the vehicle chooses BS, the curves of $\lambda_{b}=1$ and $\lambda_{b}=10$ are almost coincident, so does in the case that the vehicle chooses RSU. This shows the assumption that the influence on the coverage probability from the noise can be ignored when the BS density is large enough is reasonable. In this scene, although the BS density is low, the noise has little impact on the received SINR at the vehicle. 

### 5.2. Performance Analysis of the Proposed Game model 

In this section, the performance of the proposed caching incentive scheme and the influences of some model parameters on the performance of the game model are studied. The simulation parameters are shown in Table 2.

**Figure 7:**
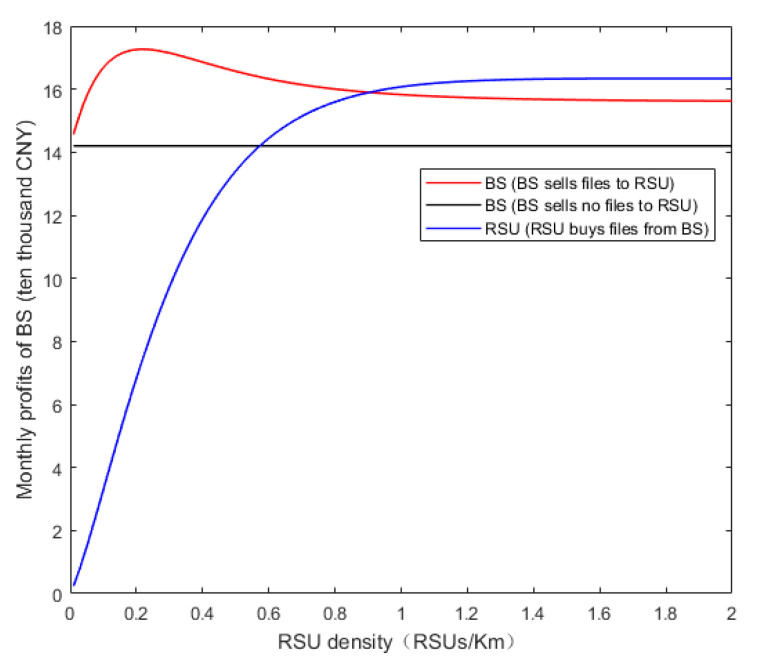
Relationships between maximum monthly profit of BS and RSU density.

The relations between the maximum monthly profits of both the BS and RSU and the RSU density are drawn in Figure 7, where the red curve, the straight line in black and the blue curve represent the monthly profits from BS selling files to RSU, BS selling no files to RSU, and RSU buying files from BS, respectively. If the BS does not sell files to the RSU, the monthly profits of BS will remain unchanged. The reason is that vehicles always download the files from the BS. However, if the BS sells files to RSU by the proposed scheme, the maximum profit of BS per month first increases and then gradually decreases with the increase of the RSU density. The reasons are twofold. First, when the RSU density is low, the increase of the RSU density will improve the coverage probability of the heterogeneous network and increase the vehicle’s willingness to download, and then the profits of both BS and RSU will go up. Second, when the RSU density is high, the coverage probability of the heterogeneous network tends to the upper limit; the game between the BS and RSU is approximate to a zero-sum game. Since increasing the RSU density leads to the vehicle tending to choose the RSU rather than the BS, the profit of BS decreases and that of RSU increases. At last, if the RSU density is high enough, the profits of both BS and RSU will not change with the increase of the RSU density. As a result, the appropriate RSU density should be chosen based on the profit and running cost per month. From the figure, compared with the condition that the BS does not sell files to the RSU, the BS can substantially increase maximum monthly profit by selling files to the RSU and the RSU also can obtain profit, which verifies the validity of the proposed scheme.

**Figure 8:**
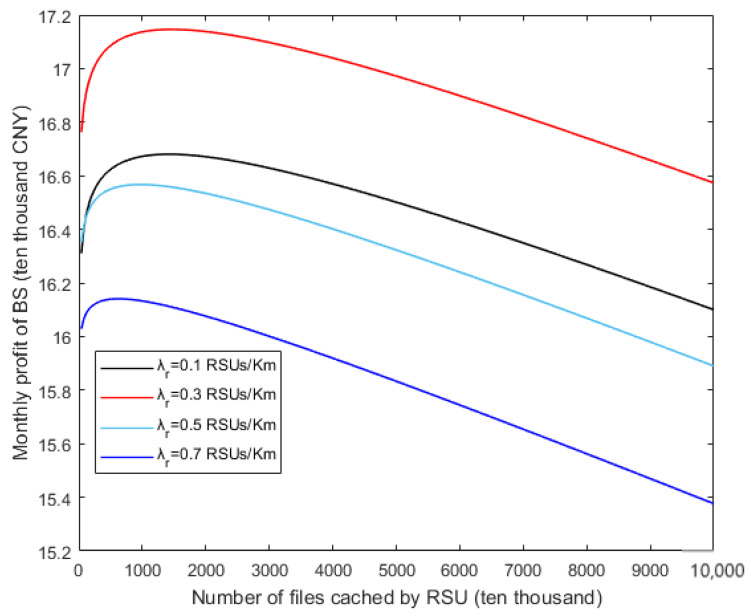
Relationships between monthly profit of BS and the number of files cached by RSU.

In Figure 8, the different curves show the relationships between the monthly profit of BS and the number of files cached by the RSU under different RSU densities. As seen from the figure, the monthly profit of the BS is a concave function w.r.t. the number of files cached by the RSU and there exists a maximum value, which is consistent with that in Section 3.3. Besides, when the different curves reach the highest point, the values of abscissa axis are different, i.e., different RSU densities correspond to different optimal number of files cached. Based on the premise that the number of files cached by RSU remains unchanged, the monthly profit of the BS first gradually increases and then continuously decreases, which is consistent with the growth trend of the red curve in Figure 7. On the contrary, under the condition that the number of files cached by RSU continues to increase, although the BS will charge more to RSU, it will cause more vehicles to select the RSU and the total profit of BS will decrease. Therefore, the BS will not allow the RSU to cache too many files.

**Figure 9:**
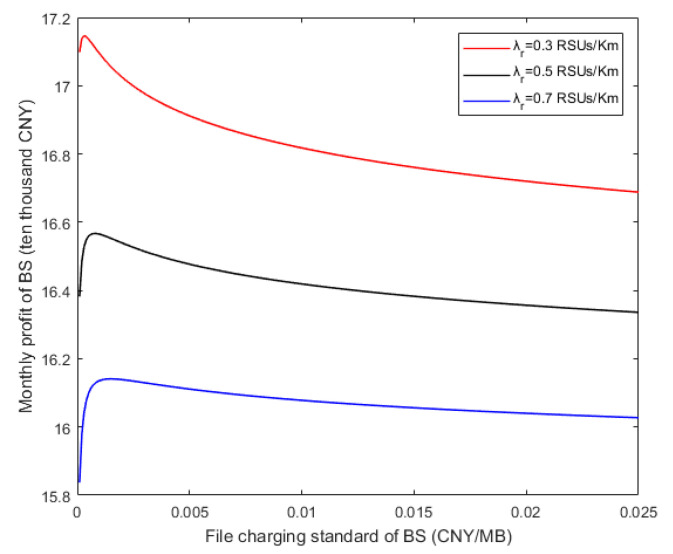
Relationships between monthly profit of BS and file pricing.

In Figure 9, the relationships between the monthly profit of BS and file charging standard of BS under different RSU densities are given. In fact, the relationship between the file charging standard of BS ($C_{f}$) and the number of cache files (h) satisfies Equation (30). That is, similar to the growth trend of the curve in Figure 8, with the increase of the file charging standard of the BS, the monthly profit of the BS first increases and then gradually decreases. In consequence, there exists the optimal file pricing maximizing the monthly profit of the BS. As a matter of fact, when the file charging standard is too low, the profit of files is very low. Then, the RSU will buy a large number of files, but it cannot bring enough profit to the BS. Inversely, when the file charging standard is too high, the RSU will be less willing to buy files from the BS, and then the BS will lose the profit from the RSU. According to the downward trends of different curves in the figure, the more the RSU density increases, the less the monthly profit of the BS decreases.

**Figure 10:**
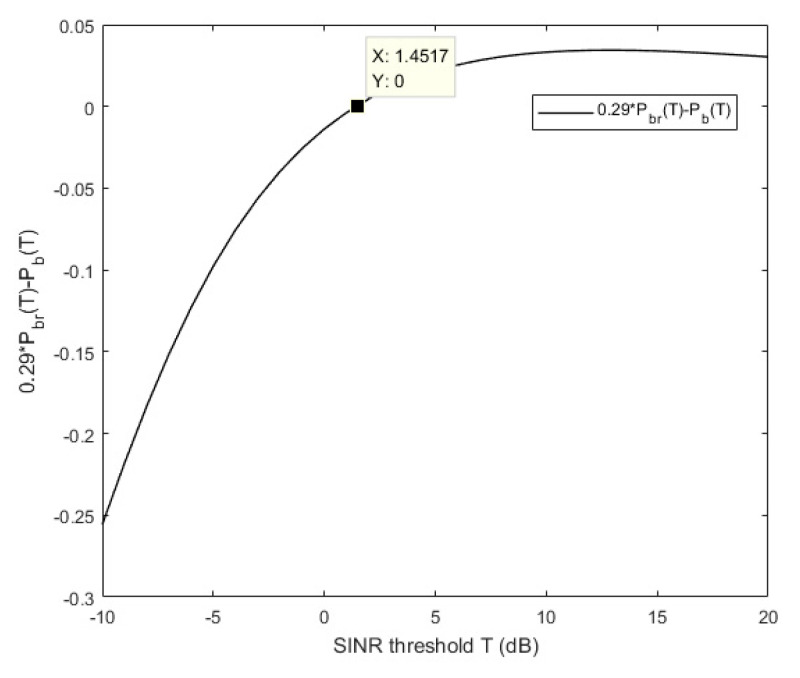
Relationships between $0.29P_{br}\left(T\right)-P_{b}\left(T\right)$ and SINR threshold T.

From Equation (36), the concavity and convexity of the profit function of the BS ($U_{b}\left(h^{*}\right)$) depend on $\left(0.29P_{br}\left(T\right)-P_{b}\left(T\right)\right)$ being positive or negative. Since the expression w.r.t. T is complicated, the curve is just plotted for the convenience of showing the relations of them in Figure 10. As seen from the figure, when −10dB≤T≤1.45 dB, $0.29P_{br}\left(T\right)-P_{b}\left(T\right)<0$; when 1.45 dB<T≤20dB, $0.29P_{br}\left(T\right)-P_{b}\left(T\right)>0$. As a result, only when the vehicle’s demand for the SINR threshold is greater than 1.452 dB, the proposed caching incentive scheme of RSU is valid. Otherwise, no matter how high the RSU density is, the proposed scheme will lower the profit of BS and the BS will not allow the RSU to cache any files from itself. In the simulation, assume the requirement of the vehicle is SINR≥20dB, so that the SINR threshold T is about 13dB. 

**Figure 11:**
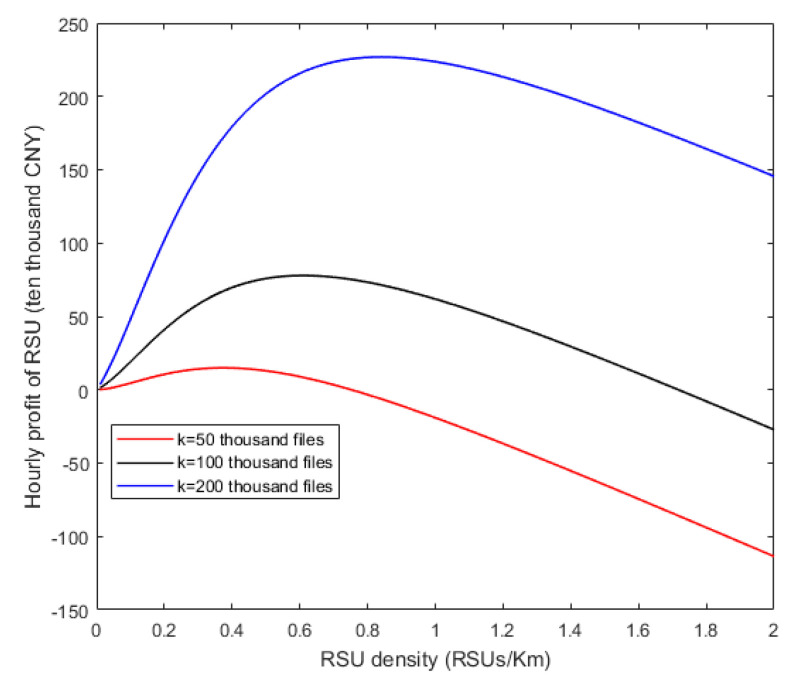
Relationships between hourly profit of RSU and RSU density.

As shown in Figure 11, the different curves represent the relationship between the hourly profit of RSU and the RSU density with different number of downloading requests of files per hour (k). If the optimal number of caching files per month of the RSU ($h^{*}$) has been determined, some RSUs need to be turned on or off during the day to adjust the active RSU density depending on the hourly traffic density for maximizing the hourly profit with considering the hourly running cost of RSUs. From the figure, the hourly profit function for the RSU is a concave function of the RSU density and the maximum is in existence. Consistent with that discussed in Section 4, different curves have different abscissa values when they reach their highest points, i.e., the density of running RSUs should be adjusted by different values of k at different hours of a day for maximizing the profit. When the profit function obtains the maximum, the profit of the RSU will decrease with the increase of the RSU density. The reason is that when the RSU density is large enough, the coverage probability of the heterogeneous network and the probability that the vehicle chooses the RSU almost cease to increase, while the running cost of RSUs increases linearly with the RSU density. As the curve at the bottom shown, when k is small, the profit from the vehicles at the RSUs is less than the running cost of RSUs. In this situation, all the RSUs should be turned off (the RSU density is 0) and no longer serve the vehicles to avoid deficit.

## 6. Conclusions

In this paper, a novel caching incentive scheme of RSUs based on Stackelberg game is proposed and its performance is analyzed in detail. The network model, file content model, and caching model of the RSU are established first, respectively. Especially in the proposed caching model, all RSUs in a region are run by the same operator. Therefore, all RSUs can be connected to the same pool by optical fiber for accessing the content purchased by any RSU for all BSs connected to the content server can sell the content to the RSUs in its coverage. Then, the expression of coverage probability of the heterogeneous network is derived based on the assumptions that the distribution of BSs submits to 2D PPP and that of RSUs submits to 1D PPP, which are verified by Monte Carlo simulation. In order to design the caching incentive scheme for RSUs based on the above-mentioned expression, the game between the BS and RSU is first modeled as a Stackelberg game and then the probabilities that moving vehicles choose the BS or RSU by the received SINR are derived. At last, the profit models of the BS and the RSU are used as the objective function for gaming and a backward introduction method is introduced to solve the Stackelberg equilibrium. Based on this, the running cost of RSUs is further considered, the operation state adjustment scheme of RSUs at different hours within a day is designed. The simulation results show that the proposed scheme can effectively increase the profits of both the BS and the RSU, and there exists the optimal density of active RSUs maximizing the hourly profit of the RSU. Future work will focus on searching for a better caching incentive method for content delivery with considering different pricing from different access nodes and trying to derive the coverage probability under the Nakagami-*m* fading channel.

## Figures and Tables

**Table 1:** Simulation parameters.

Parameter	Value
Radius of base station distribution area	R=10 km
Transmission power of BS	$P_{1}=50\;W$[35]
Transmission power of RSU	$P_{0}=1\;W$[36]
Bandwidth	$B_{0}=10\;\mathrm{MHz}$
Power spectral density of noise	$N_{0}=-174\;\mathrm{dBm}/\mathrm{Hz}$
Path loss exponent	α=4

**Table 2:** Simulation parameters.

Parameter	Value
Zipf distribution parameter	Ω=1
Threshold of effective coverage	T=13 dB
Transmission power of RSU	$P_{0}=1\;W$
Bandwidth	$B_{0}=10\;\mathrm{MHz}$
Power spectral density of noise	$N_{0}=-174\;\mathrm{dBm}/\mathrm{Hz}$
Path loss exponent	α=4
Size of single content file	L=1 MB
Total number of files	$F=10^{13}$
Euler’s constant	γ=0.5772
Number of file downloading requests from vehicles in one month	$K=10^{8}$
Number of file downloading requests from vehicles within an hour	$k=10^{5}$
BSs and RSUs charge vehicles for unit traffic pricing	$C_{v}=$ 0.01 CNY/MB
Pricing difference determined by BS	$C_{m}=10^{-4}$ CNY/MB
Hourly running cost for single RSU	$C_{r}=10$ CNY/h
Length of the road	D=10 km

## Appendix A

Take the tagged vehicle as the origin o. Since the distance between the origin and the nearest RSU is r, none of the interfering RSUs can be closer than r. Therefore, the interference is a standard *M*/*M*; shot noise created by 1D PPP at origin o [37,38]. As seen in Equation (1), $I_{r}$ is the cumulative interference power from the other RSUs. Let $L_{I_{r}}\left(s\right)$ be the Laplace transform of random variable $I_{r}$. By using the definition of the Laplace transform, $L_{I_{r}}\left(s\right)$ can be expressed as

(A1) $$\begin{array}{ll} L_{I_{r}}\left(s\right) & =E_{I_{r}}\left(e^{-sI_{r}}\right)=E_{\Phi ,g}\left[exp\left(-s\underset{i\in \Phi \backslash \left\{r_{o}\right\}}{\sum }g_{i}R_{i}^{-\alpha }\right)\right]\\ & =E_{\Phi ,g}\left[\underset{i\in \Phi \backslash \left\{r_{o}\right\}}{\prod }exp\left(-sg_{i}R_{i}^{-\alpha }\right)\right]\\ & \frac{\underline{\left(a\right)}}{\;}E_{\Phi }\left[\underset{i\in \Phi \backslash \left\{r_{o}\right\}}{\prod }E_{g}\left[exp\left(-sg_{i}R_{i}^{-\alpha }\right)\right]\right]\\ & =exp\left(-\lambda \int_{r}^{\infty }\left(1-E_{g}\left[exp\left(-sg_{i}v^{-\alpha }\right)\right]\right)dv\right) \end{array}$$

where $R_{i}$ denotes the distance between the origin o and the *i*th interference RSU with fading channel coefficient $g_{i}$, and (a) follows from the i.i.d. distribution of $g_{i}$. Φ denotes RSUs obeying PPP and $\Phi \backslash \left\{r_{o}\right\}$ denotes RSUs that the distance between the origin and RSU is larger than r. And the last step follows from the probability generating functional of the PPP [39], i.e.,

(A2) $$E\left[\underset{x\in \Phi }{\prod }f\left(x\right)\right]=exp\left(-\lambda \int_{R^{d}}^{}\left(1-f\left(x\right)\right)dx\right)$$

where d denotes the dimensionality of PPP. When Φ is 1D PPP, d=1. Since the distances between the tagged vehicle and the interfering RSUs are all larger than r, the integration limits of Equation (A2) are from r to ∞. Since the channel is Rayleigh fading, i.e., $g_{i}\sim exp\left(\mu \right)$, Equation (A1) can be simplified as

(A3) $$\begin{array}{ll} L_{I_{r}}\left(s\right) & =E_{\Phi }\left[\underset{i\in \Phi \backslash \left\{r_{o}\right\}}{\prod }E_{g}\left[exp\left(-sg_{i}R_{i}^{-\alpha }\right)\right]\right]\\ & =E_{\Phi }\left[\underset{i\in \Phi \backslash \left\{r_{o}\right\}}{\prod }\frac{\mu }{\mu +sR_{i}^{-\alpha }}\right]\\ & =exp\left(-\lambda \int_{r}^{\infty }\left(1-\frac{\mu }{\mu +sv^{-\alpha }}\right)dv\right) \end{array}$$

## Appendix B

If the profit function of the RSU, i.e., $U_{r}\left(\lambda_{r}\right)$, is a strictly concave function w.r.t. $\lambda_{r}$, there exists the optimal value $\lambda_{r}^{*}$ maximizing $U_{r}\left(\lambda_{r}\right)$. As seen from Equation (44), if $\left[P_{b}\left(T\right)+\left[1-P_{b}\left(T\right)\right]P_{r}\left(T,\lambda_{r}\right)\right]\cdot G\left(\lambda_{r}\right)$ is concave, $U_{r}\left(\lambda_{r}\right)$ is also concave. According to the addition rule of concavity and convexity [40,41], if both $G\left(\lambda_{r}\right)$ and $P_{r}\left(T,\lambda_{r}\right)\cdot G\left(\lambda_{r}\right)$ are concave, $\left[P_{b}\left(T\right)+\left[1-P_{b}\left(T\right)\right]P_{r}\left(T,\lambda_{r}\right)\right]\cdot G\left(\lambda_{r}\right)$ is also concave. Therefore, the proof of concavity and convexity of $U_{r}\left(\lambda_{r}\right)$ is equivalent to that of $G\left(\lambda_{r}\right)$ and $P_{r}\left(T,\lambda_{r}\right)\cdot G\left(\lambda_{r}\right)$.

For convenience, let $r=\frac{v}{\lambda_{r}}$, $P_{r}\left(T,\lambda_{r}\right)$ can be calculated by

(A4) $$\begin{array}{cc} P_{r}\left(T,\lambda_{r}\right) & =\int_{0}^{\infty }exp\left(-\lambda_{r}\cdot r\left[1+T^{\frac{1}{4}}\int_{T^{-\frac{1}{4}}}^{\infty }\left(\frac{1}{1+x^{4}}\right)dx\right]-\mu_{r}Tr^{4}\sigma^{2}\right)\lambda_{r}dr\\ & =\int_{0}^{\infty }exp\left(-v\cdot \left[1+T^{\frac{1}{4}}\int_{T^{-\frac{1}{4}}}^{\infty }\left(\frac{1}{1+x^{4}}\right)dx\right]-\mu_{r}T\sigma^{2}\lambda_{r}^{-4}v^{4}\right)dv\\ & =\int_{0}^{\infty }exp\left(-A_{r}\left(T\right)\cdot v-B_{r}\left(T\right)\cdot \lambda_{r}^{-4}v^{4}\right)dv \end{array}$$

where $A_{r}\left(T\right)=1+T^{\frac{1}{4}}\int_{T^{-\frac{1}{4}}}^{\infty }\left(\frac{1}{1+x^{4}}\right)dx$ and $B_{r}\left(T\right)=\mu_{r}T\sigma^{2}$. No matter what the value of T is, both of and $B_{r}\left(T\right)$ are positive. Substitute (A4) into expressions of $G\left(\lambda_{r}\right)$ and $P_{r}\left(T,\lambda_{r}\right)\cdot G\left(\lambda_{r}\right)$, the following Equations (A5) and (A6) can be obtained.

(A5) $$G\left(\lambda_{r}\right)=\int_{0}^{\infty }f\left(t\right)\cdot \int_{0}^{\infty }exp\left(-A_{r}\left(t\right)\cdot v-B_{r}\left(t\right)\cdot \lambda_{r}^{-4}v^{4}\right)dvdt$$

(A6) $$P_{r}\left(T,\lambda_{r}\right)\cdot G\left(\lambda_{r}\right)=\int_{0}^{\infty }e^{-A_{r}\left(T\right)v-B_{r}\left(T\right)\lambda_{r}^{-4}v^{4}}dv\cdot \int_{0}^{\infty }f\left(t\right)\int_{0}^{\infty }e^{-A_{r}\left(t\right)v-B_{r}\left(t\right)\lambda_{r}^{-4}v^{4}}dvdt$$

According to literature [42], it is easy to obtain

(A7) $$\frac{d}{dt}\int_{b\left(t\right)}^{a\left(t\right)}f\left(x,t\right)dx=\int_{b\left(t\right)}^{a\left(t\right)}\frac{\partial f\left(x,t\right)}{\partial t}dx+f\left[a\left(t\right),t\right]\frac{da\left(t\right)}{dt}-f\left[b\left(t\right),t\right]\frac{db\left(t\right)}{dt}$$

where t is the parameter and f(x,t) is a function containing *t*;. Then, by taking advantage of Equation (A7), the first and second partial derivatives of $G\left(\lambda_{r}\right)$ can be expressed by

(A8) $$\frac{\partial G\left(\lambda_{r}\right)}{\partial \lambda_{r}}=4\lambda_{r}^{-5}\int_{0}^{\infty }[B_{r}\left(t\right)\cdot f\left(t\right)\cdot \int_{0}^{\infty }v^{4}e^{-A_{r}\left(t\right)v-B_{r}\left(t\right)\lambda_{r}^{-4}v^{4}}dv]dt$$

(A9) $$\frac{\partial^{2}G\left(\lambda_{r}\right)}{\partial \lambda_{r}{}^{2}}=-4\lambda_{r}^{-6}\int_{0}^{\infty }[A_{r}\left(t\right)B_{r}\left(t\right)f\left(t\right)\int_{0}^{\infty }v^{5}e^{-A_{r}\left(t\right)v-B_{r}\left(t\right)\lambda_{r}^{-4}v^{4}}dv]dt<0$$

Therefore, $G\left(\lambda_{r}\right)$ is a concave function w.r.t. $\lambda_{r}$.

By utilizing Equation (A7) again, (A10) can be obtained, which is expressed as

(A10) $$\begin{array}{l} \frac{\partial^{2}\left[P_{r}\left(T,\lambda_{r}\right)\cdot G\left(\lambda_{r}\right)\right]}{\partial \lambda_{r}{}^{2}}=2\frac{\partial P_{r}\left(T,\lambda_{r}\right)}{\partial \lambda_{r}}\cdot \frac{\partial G\left(\lambda_{r}\right)}{\partial \lambda_{r}}+\frac{\partial^{2}P_{r}\left(T,\lambda_{r}\right)}{\partial \lambda_{r}{}^{2}}\cdot G\left(\lambda_{r}\right)+P_{r}\left(T,\lambda_{r}\right)\cdot \frac{\partial^{2}G\left(\lambda_{r}\right)}{\partial \lambda_{r}{}^{2}}\\ =32B_{r}\left(T\right)\lambda_{r}^{-10}\int_{0}^{\infty }e^{-A_{r}\left(T\right)v-B_{r}\left(T\right)\lambda_{r}^{-4}v^{4}}dv\cdot \int_{0}^{\infty }[B_{r}\left(t\right)f\left(t\right)\int_{0}^{\infty }v^{4}e^{-A_{r}\left(t\right)v-B_{r}\left(t\right)\lambda_{r}^{-4}v^{4}}dv]dt\\ \begin{array}{cc} & -4A_{r}\left(T\right)B_{r}\left(T\right)\lambda_{r}^{-6}\int_{0}^{\infty }v^{5}e^{-A_{r}\left(T\right)v-B_{r}\left(T\right)\lambda_{r}^{-4}v^{4}}dv\cdot \int_{0}^{\infty }\left[f\left(t\right)\int_{0}^{\infty }e^{-A_{r}\left(t\right)v-B_{r}\left(t\right)\lambda_{r}^{-4}v^{4}}dv\right]\;dt \end{array}\\ \begin{array}{cc} & -4\lambda_{r}^{-6}\int_{0}^{\infty }e^{-A_{r}\left(T\right)v-B_{r}\left(T\right)\lambda_{r}^{-4}v^{4}}dv\cdot \int_{0}^{\infty }[A_{r}\left(t\right)B_{r}\left(t\right)f\left(t\right)\int_{0}^{\infty }v^{5}e^{-A_{r}\left(t\right)v-B_{r}\left(t\right)\lambda_{r}^{-4}v^{4}}dv]dt \end{array}\\ =4\lambda_{r}^{-6}\int_{0}^{\infty }e^{-A_{r}\left(T\right)v-B_{r}\left(T\right)\lambda_{r}^{-4}v^{4}}dv\cdot \\ \begin{array}{cc} & \left\{\int_{0}^{\infty }[B_{r}\left(t\right)f\left(t\right)\cdot (8B_{r}\left(T\right)\lambda_{r}^{-4}\int_{0}^{\infty }v^{4}e^{-A_{r}\left(t\right)v-B_{r}\left(t\right)\lambda_{r}^{-4}v^{4}}dv-A_{r}\left(t\right)\int_{0}^{\infty }v^{5}e^{-A_{r}\left(t\right)v-B_{r}\left(t\right)\lambda_{r}^{-4}v^{4}}dv)]dt\right\} \end{array} \end{array}$$

The positive and negative of the above Equation (A10) is determined by

(A11) $$\begin{array}{l} 8B_{r}\left(T\right)\lambda_{r}^{-4}\int_{0}^{\infty }v^{4}e^{-A_{r}\left(t\right)v-B_{r}\left(t\right)\lambda_{r}^{-4}v^{4}}dv-A_{r}\left(t\right)\int_{0}^{\infty }v^{5}e^{-A_{r}\left(t\right)v-B_{r}\left(t\right)\lambda_{r}^{-4}v^{4}}dv\\ =8B_{r}\left(T\right)\lambda_{r}^{-4}\int_{0}^{1}v^{4}e^{-A_{r}\left(t\right)v-B_{r}\left(t\right)\lambda_{r}^{-4}v^{4}}dv-A_{r}\left(t\right)\int_{0}^{1}v^{5}e^{-A_{r}\left(t\right)v-B_{r}\left(t\right)\lambda_{r}^{-4}v^{4}}dv\\ +8B_{r}\left(T\right)\lambda_{r}^{-4}\int_{1}^{\infty }v^{4}e^{-A_{r}\left(t\right)v-B_{r}\left(t\right)\lambda_{r}^{-4}v^{4}}dv-A_{r}\left(t\right)\int_{1}^{\infty }v^{5}e^{-A_{r}\left(t\right)v-B_{r}\left(t\right)\lambda_{r}^{-4}v^{4}}dv \end{array}$$

Then, by enlarging the second and third terms in Equation (A11), it can be expressed as

(A12) $$\begin{array}{l} 8B_{r}\left(T\right)\lambda_{r}^{-4}\int_{0}^{1}v^{4}e^{-A_{r}\left(t\right)v-B_{r}\left(t\right)\lambda_{r}^{-4}v^{4}}dv-A_{r}\left(t\right)\int_{0}^{1}v^{5}e^{-A_{r}\left(t\right)v-B_{r}\left(t\right)\lambda_{r}^{-4}v^{4}}dv\\ +8B_{r}\left(T\right)\lambda_{r}^{-4}\int_{1}^{\infty }v^{4}e^{-A_{r}\left(t\right)v-B_{r}\left(t\right)\lambda_{r}^{-4}v^{4}}dv-A_{r}\left(t\right)\int_{1}^{\infty }v^{5}e^{-A_{r}\left(t\right)v-B_{r}\left(t\right)\lambda_{r}^{-4}v^{4}}dv\\ \leq 8B_{r}\left(T\right)\lambda_{r}^{-4}\int_{0}^{1}v^{4}e^{-A_{r}\left(t\right)v-B_{r}\left(t\right)\lambda_{r}^{-4}v^{4}}dv-A_{r}\left(t\right)\int_{0}^{1}v^{4}e^{-A_{r}\left(t\right)v-B_{r}\left(t\right)\lambda_{r}^{-4}v^{4}}dv\\ +8B_{r}\left(T\right)\lambda_{r}^{-4}\int_{1}^{\infty }v^{5}e^{-A_{r}\left(t\right)v-B_{r}\left(t\right)\lambda_{r}^{-4}v^{4}}dv-A_{r}\left(t\right)\int_{1}^{\infty }v^{5}e^{-A_{r}\left(t\right)v-B_{r}\left(t\right)\lambda_{r}^{-4}v^{4}}dv\\ =[8B_{r}\left(T\right)\lambda_{r}^{-4}-A_{r}\left(t\right)]\cdot (\int_{0}^{1}v^{4}e^{-A_{r}\left(t\right)v-B_{r}\left(t\right)\lambda_{r}^{-4}v^{4}}dv+\int_{1}^{\infty }v^{5}e^{-A_{r}\left(t\right)v-B_{r}\left(t\right)\lambda_{r}^{-4}v^{4}}dv) \end{array}$$

Since $B_{r}\left(T\right)\lambda_{r}^{-4}=\mu_{r}T\sigma^{2}\lambda_{r}^{-4}$ ($\sigma^{2}=B_{0}\cdot N_{0}=-104\mathrm{dBm}\approx 3.98\times 10^{-14}W$), the RSU density $\lambda_{r}$ generally is not less than 0.5 RSUs/km, and $A_{r}\left(t\right)=1+t^{\frac{1}{4}}\int_{t^{-\frac{1}{4}}}^{\infty }\left(\frac{1}{1+x^{4}}\right)dx>1$, the two expressions $B_{r}\left(T\right)\lambda_{r}^{-4}\ll A_{r}\left(t\right)$ and $8B_{r}\left(T\right)\lambda_{r}^{-4}-A_{r}\left(t\right)<0$ can be easily obtained. Therefore, the value of Equation (A12) is less than 0 and then $\frac{\partial^{2}\left[P_{r}\left(T,\lambda_{r}\right)\cdot G\left(\lambda_{r}\right)\right]}{\partial \lambda_{r}{}^{2}}<0$. Therefore, both $G\left(\lambda_{r}\right)$ and $P_{r}\left(T,\lambda_{r}\right)\cdot G\left(\lambda_{r}\right)$ are the concave functions w.r.t. $\lambda_{r}$, and then the one-hour profit function of the RSU $U_{r}\left(\lambda_{r}\right)$ is also concave.
